# FIDChain: Federated Intrusion Detection System for Blockchain-Enabled IoT Healthcare Applications

**DOI:** 10.3390/healthcare10061110

**Published:** 2022-06-15

**Authors:** Eman Ashraf, Nihal F. F. Areed, Hanaa Salem, Ehab H. Abdelhay, Ahmed Farouk

**Affiliations:** 1 Department of Electronics and Communications Engineering, Faculty of Engineering, Delta University for Science and Technology, Gamasa 35712, Egypt; hana.salem@deltauniv.edu.eg; 2 Department of Electronics and Communications Engineering, Faculty of Engineering, Mansoura University, Mansoura 35516, Egypt; nahoolaf@mans.edu.eg (N.F.F.A.); ehababdelhay@mans.edu.eg (E.H.A.); 3 Centre for Photonics and Smart Materials, Zewail City of Science and Technology, Giza 12578, Egypt; 4 Department of Computer Science, Faculty of Computers and Artificial Intelligence, South Valley University, Hurghada 84511, Egypt; ahmed.farouk@sci.svu.edu.eg

**Keywords:** IoT, intrusion detection, healthcare security, federated learning, blockchain, machine learning

## Abstract

Recently, there has been considerable growth in the internet of things (IoT)-based healthcare applications; however, they suffer from a lack of intrusion detection systems (IDS). Leveraging recent technologies, such as machine learning (ML), edge computing, and blockchain, can provide suitable and strong security solutions for preserving the privacy of medical data. In this paper, FIDChain IDS is proposed using lightweight artificial neural networks (ANN) in a federated learning (FL) way to ensure healthcare data privacy preservation with the advances of blockchain technology that provides a distributed ledger for aggregating the local weights and then broadcasting the updated global weights after averaging, which prevents poisoning attacks and provides full transparency and immutability over the distributed system with negligible overhead. Applying the detection model at the edge protects the cloud if an attack happens, as it blocks the data from its gateway with smaller detection time and lesser computing and processing capacity as FL deals with smaller sets of data. The ANN and eXtreme Gradient Boosting (XGBoost) models were evaluated using the BoT-IoT dataset. The results show that ANN models have higher accuracy and better performance with the heterogeneity of data in IoT devices, such as intensive care unit (ICU) in healthcare systems. Testing the FIDChain with different datasets (CSE-CIC-IDS2018, Bot Net IoT, and KDD Cup 99) reveals that the BoT-IoT dataset has the most stable and accurate results for testing IoT applications, such as those used in healthcare systems.

## 1. Introduction

IoT plays a significant role in the development of the healthcare industry. Furthermore, it has become a considerable and important source of medical data as the physical devices collect the vital signs using numerous sensors and share the real-time data with the medical team by connecting to the internet. Shaikh et al. [1] proposed a system that makes use of embedded wearable sensors to monitor health parameters remotely, storing the analyzed data on the cloud, and automatically sending results of the analysis to a doctor when there is a critical condition. The proposed system minimizes health costs by reducing the number of times the doctor visits. Rohokale et al. [2] proposed a health monitoring system for controlling human health parameters, and applied this system in a safe motherhood program application. The results revealed that the system saves about 57% energy, and it supports the concept of green IoT communication, as well as enhancing throughput. The storage of medical big data in information systems that are based on cloud-client servers are suffering from single-point failure, and the controlling of data resources in a centralized manner leads to privacy leakage [3]. Li et al. [4] provides a solution to these problems using the advances of blockchain technology to support the healthcare system. Blockchain is a decentralized system that allows transaction transmission and storage under the roles that are listed in consensus algorithm and smart contracts with no central authority in a distributed ledger [5]. The blockchain framework provides different features, including decentralization, privacy, and security. The blockchain storage uses cryptographic keys to secure the user identity. Aujla et al. [6] presented a blockchain-based edge system for data tampering and privacy preserving of the patient’s medical records. The system analysis shows the effectiveness of it in terms of the block preparation time, header generation time, tensor reduction ratio, and approximation error. The adoption of blockchain technology with the IoT has a lot of benefits, such as immutability, transparency, and data provenance [7]. There are many blockchain-based IoT applications in the healthcare industry, such as electronic medical records management [8], remote patient monitoring [9], drug traceability [10], and infectious-disease fighting [11].

It is assured that IoT devices are easily hackable and could be controlled remotely to form IoT-based botnets [12,13]. These attacks and botnets cause the leakage of sensitive information, infraction, and infringement in the wider IoT-enabled system [14]. Some of the most common attacks in IoT systems include distributed denial-of-service (DDoS), denial of service (DOS), ransom ware, and botnet attacks [15]. Therefore, there is considerable research going on in the area of security and authentication issues for IoT-based healthcare systems. There is a rapid growth in the research of ML-based healthcare applications. Some ML models have been used in the diagnosis of diseases [16], while others have been used in IDS for security issues. The need for IDS techniques is vital because of the resource-constraint considerations in IoT devices [17]. Mohapatra et al. [18] proposed a cloud-based model that transfers and stores patient data over a cloud, and its security system involves approving user authentication by barcode sensor. The doctor can verify patient data securely and give his valuable feedback. The cloud-computing technology provides a backbone server called cloud to process and store data used to train the ML model. Doriguzzi-Corin et al. [19] proposed LUCID, a convolutional neural network (CNN)-based IDS, because of its ability of pattern recognition to classify benign traffic from DDoS attacks. Latif et al. [20] presented a novel random neural network (RaNN) for predicting attacks, such as DoS, malicious operation, malicious control, data type probing, spying, and scan. The presented RaNN was compared with the traditional ANN, the support vector machine (SVM), and the decision tree (DT). In addition, the proposed algorithm RaNN achieves higher attack detection accuracy by an average of 5.65% compared to that the others. However, traditional cloud-computing models suffer from high latency and losses that are due to the crowded backbone network [21]. Edge computing is used to address the limitations of cloud computing in supporting IoT applications [22,23]. Edge computing is an inventive technology that processes data to the edge of the network closer to the end-user rather than performing this previously in the core network [24]. An edge-assisted IoT layer provides lower latency, more flexible access, protection of data privacy, and enhanced quality of service [25].

Most of the previous IDS was deployed in the core cloud layer, which did not meet the real security needs for protecting real-time healthcare monitoring data. If the intrusion is not detected in time, it will cause incalculable damage to the applications and devices in the IoT [26]. To train the central model, the privacy data must be uploaded to a central entity; however, the transfer of personal data to a centralized entity affects the privacy. This introduces a single point of failure that affects the integrity of data and quality of services. The centralized IDS is time-consuming. Collecting diversified data types (e.g., text, audio, video, and AR/VR) in the 5G/6G network is very costly [27]. Due to the previous issues encountered in the research on security of healthcare systems, such research is still in the development stage and the applicability of the intrusion detection technology has raised higher requirements. Although ML and DL technologies have significant contributions in solving real-word problems, they have various constraints. McMahan et al. [28] with the Google team presented an alternative technique to the centralized learning of deep networks by leaving the training data distributed on the mobile devices, training mobile models locally at the edge layer, aggregating locally computed updates to the server, learning the global model, and, finally, broadcasting learning updates to local models. They called this approach “federated learning (FL)”. This method preserves the privacy of the locally trained data, which is necessary for various applications in the healthcare sector. Zhao et al. [29] proposed a multitask deep neural network in FL (MT-DNN-FL). For evaluation, the datasets of CICIDS2017, ISCXVPN2016, and ISCXT were used. The results showed that the proposed algorithm has a good detection rate in the multi-tasks and decreases the overhead of training time more than centralized training. The proposed model needs to optimize the DNN structure to cope with the restrictions of IoT devices. Rajendran et al. [30] proposed two FL models with ANN and logistic regression (LR) for protecting patient data privacy and security in healthcare systems. However, due to lower complexity of LR and lack of epochs, FL do not have the potential to improve the performance of the models. Compared with ANN models, FL performs better in accuracy and privacy. Rieke et al. [31] discussed the impact of FL on the future of digital healthcare by presenting the issues of medical sensitive data privacy without the need to exchange or centralize datasets. Despite the advances of the FL, it has a big challenge, which is the poisoning attack for the training data and the global model through poisoning aggregated weights, which is some kind of man-in-the-middle attack. Using model reverse engineering with the help of aggregated weights of local models, the private data could be compromised [32]. In [33], the authors presented a novel FL poisoning backdoor into the aggregated data for IDS local models by changing training datasets to incorrectly classify malicious traffic. The data poisoning attack occurs when the attacker poisons the training data by inserting small amounts of backdoor malicious data. The work showed the effectiveness of the attack toward the damage of the data. Bagdasaryan et al. [34] proposed a model poisoning attack that is more powerful than the data poisoning attack. The proposed poisoning attack affects aggregated model updates that train the global model after averaging. Zhang et al. [35] proposed generative adversarial networks (GAN) to poison the model while training and the private data is cloned. After training, the label of the generated model data is poisoned, which increases the amount of effect on the global models.

From the above research, there is a need for more feasible solutions and studies to tackle FL poisoning attacks. Nguyen et al. [33] stated that the solution of averaging out poisoned updates by scaling down the models with high amplitudes of updates could damage the performance and negatively impact the model’s main task. The incorporation of new technologies with the FL, such as modern communication protocols, encryption standards, blockchain, and lightweight DL, could provide a good solution [27]. Much research stated the benefits of leveraging blockchain technology for IoT systems in healthcare applications. Alkadi et al. [36] proposed a deep blockchain framework for IDS-based IoT networks to identify cyberattacks in the centralized cloud environment. The proposed framework achieves better performance against inference and data poisoning attacks, but it still has privacy preserving concerns that are due to centralized learning. In this study, we propose a framework to integrate the blockchain technology and the FL-based IDS network into IoT security in healthcare systems. The framework can maintain secure transaction records of the local model weights, help in selective model aggregation, and effectively protect the system from poisoning attacks.

The contributions of our proposed system are:

- Proposing an IDS model for preventing attacks on a healthcare system using lightweight detection model to cope with insufficient memory space and resource-constraint considerations of edge nodes. The ANN was selected because of its advantages, as it does not have any restrictions on datasets and its distribution and has better performance with the heterogeneity of data in IoT devices, such as ICU in healthcare systems.
- Introducing an edge-cloud IDS architecture in a federated way to prevent the centralized manner problems, such as single point of failure, and preserve the privacy of the local trained data, which is necessary for various applications in the healthcare sector. Besides that, applying the detection model at the edge layer near the source of the attacks makes the detection response quicker, as well as reduces the cloud’s workload.
- Integrating blockchain technology with FL manner to store the local weights for updating the global model, which protects the system from poisoning attacks and provides full transparency and immutability over the distributed training process.

The rest of the paper is organized as follows. Section 2 gives the description of the proposed system and its layered architecture, algorithm of FIDChain, and its detection model. Section 3 provides the preprocessing steps of the datasets, the evaluation results of FIDChain, evaluation of blockchain with the federated model, discussion and comparison with the state of the art of different related studies then using different datasets. Finally, Section 4 gives the conclusion and directions for future work.

## 2. Materials and Methods

### 2.1. Proposed System

**Figure 1:**
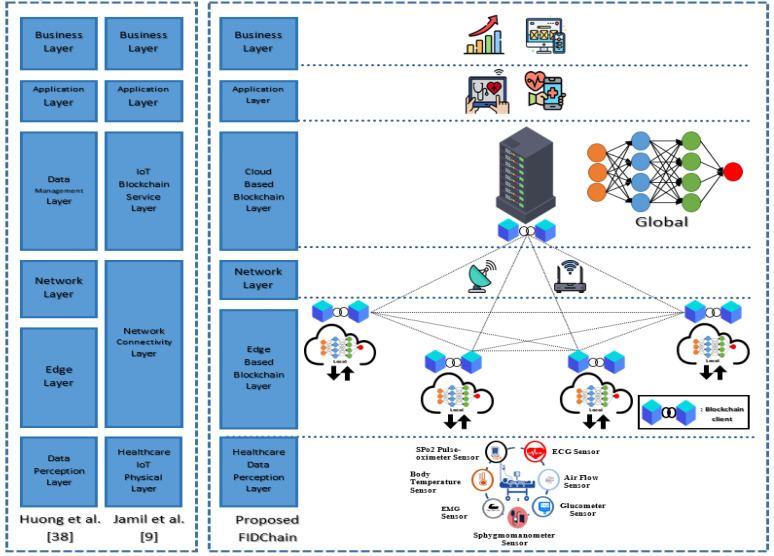
Layered architecture of the proposed system FIDChain [9,38].

According to the required functions of IoT applications, there are different system architectures [37]. Figure 1 presents a comparison of the layered architectures between our proposed system and those proposed in [9,38]. The proposed system architecture in [9] is composed of a physical layer, network connectivity layer, IoT blockchain cloud layer, application layer, business layer. The system is proposed for monitoring patient vital signs using smart contracts based on blockchain without any classification for these data using machine learning algorithm. The system did not discuss the security issues of intrusion detection. The system is centralized, suffering from single point of failure and compromising medical information as it sends it to a central server.

**Figure 2:**
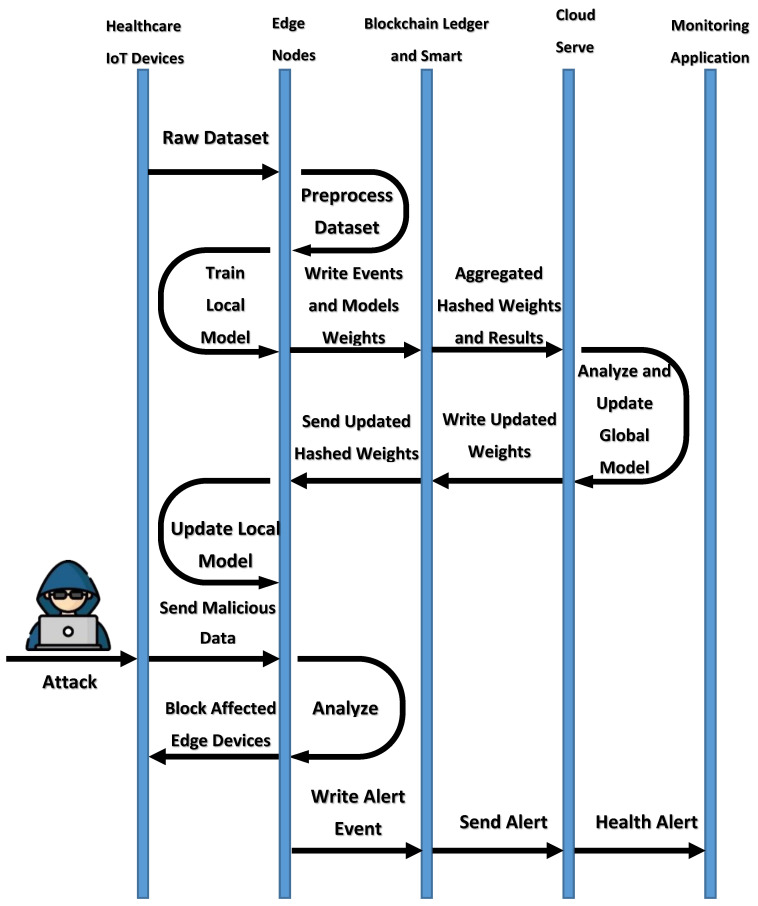
Flow execution of the proposed FIDChain system.

**Figure 3:**
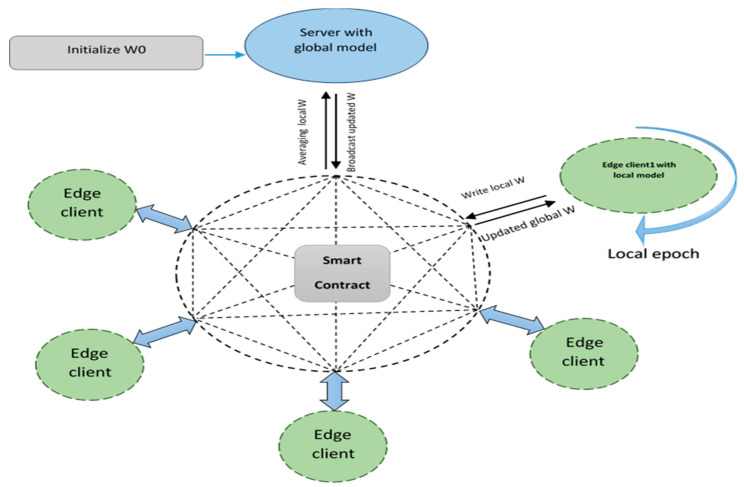
Diagram of the FIDChain aggregation of weights into the blockchain network.

The proposed system architecture in [38] is composed of a data perception layer, edge layer, network layer, data management (cloud layer), application layer, and business layer. The proposed system is a multi-attack edge layer detection mechanism in a federated manner. The intrusion detection system did not discuss the poisoning attacks problem during the sending the weights for aggregation to the server. The accuracy results have an average of 92%, which needs more enhancement. The architecture layers of the proposed FIDChain system are described as follows:

- Healthcare data perception layer: this contains ICU IoT devices with sensors. ICU case devices could be classified into two categories: room environmental monitoring devices and patient health monitoring devices.
- Edge-based blockchain layer: this consists of IoT gateways. Each gateway contains some healthcare sensing devices. There is no global internal protocol for physical healthcare sensors; therefore, a lot of network access protocols were supported by the gateways. An IoT gateway is responsible for performing a multi-attack detection. At the edge server (ES), a lightweight IDS was developed to normalize their data and detect several ANN-based attacks. The proposed module will be developed in the FL mode and trained in the edge layer so as to protect the cloud or other resources if a particular attack happens, as it blocks the data from its gateway. The detection time of intrusion will be smaller as the attack resources are near. In addition, there will be lesser computing and processing capacity because the FL model deals with smaller sets of data. After the module learning process is completed, the weights of each local model will be sent to a blockchain-distributed ledger and stored in chained blocks that connect gateway nodes with a server node in the next cloud layer. These chained blocks will be further used for aggregation and averaging purposes. Finally, that chain is protected using a cryptographic hash function that connects the blocks together in the chain and, consequently, it cannot be manipulated or changed, as it operates by consensus algorithms (smart contracts). The flow execution of the overall system is described in Figure 2. The proposed FIDChain model provides a solution to poisoning attacks, which is one of the most important challenges facing FL. In which, every ES acquires the values of the updated weights and encrypts the collected data and generates the corresponding signature using its own secret key. Then ES aggregates the ciphertext and submits it with the signature to the activated blockchain layer controlled by a smart contract, keeping control of data privacy and data integrity. When receiving the data of all ESs, smart contract verifies the validity of these messages using the ES’ public keys and stored data as blockchain hashed blocks under smart contract rules. In turn, the central public cloud center (CPCC) can fetch the stored blocks from the activated blockchain, the CPCC can retrieve the aggregated plaintext using its own secret key. In general applications of IoT edge computing, the communications between CPCC and ESs, and ES and the corresponding local model are both two-way. As in terminal edge computing-based data storage, local model can both upload and download the data to or from CPCC via blochchain network and ES. Algorithm 1 gives the pseudocode for the FIDChain.
- Network layer: this is responsible for securing transaction of data from the lower layer to the higher layer. It is considered as the connectivity layer that aims to provide routing management.
- Cloud-based blockchain layer: the cloud is in charge of aggregated weights in the blockchain ledger from the ESs, taking average weights and updating the global weights of the ANN algorithm. Periodically, the cloud sends the aforementioned updated weights to all gateways for updating local models’ weights to protect the network efficiently. Figure 3 describes the diagram of the FIDChain aggregation of local and global weights into the blockchain network.
- Application layer: this is responsible for monitoring healthcare vital signs.
- Business layer: this helps managers of the whole healthcare application service to create business models, flow charts, and executive reports based on analyzed and received data from lower layers.

**Algorithm 1: FIDChain****1:**Input: N is the node number atIDSFChain; g is global round; C is local epochs; M is the local batch size; K no. of edge gateways; n_k_ is size of data partition of edge gateway k; and η is the learning rate.**2:**Output: updated weights W**3:**Procedure Server_ Node1 _Update:**4:**Initialize w0**5:**//IDSFChain is name of blockchain network**6:**Node1: creates IDSFChain**7:**Node1: connects to IDSFChainwith ip address**8:**For each local edge from 1 to C do**9:**  //IDSFChainnode no. = edge node no. + 1**10:**N = C + 1**11:**Node1: grant mining of other nodes (sending and writing)**12:**Node_N_: connects to IDSFChain with ip address**13:**End for**14:**Node1: publish initial weights w0**15:**For each global epoch g = 1, 2, … do**16:**For each Node_N_ ∈ IDSFChain N from 2 to (k + 1) in parallel **do****17:**$W_{g+1}^{K}$← Edge_Nodes_Update (Node_N_, w_g_)**18:**End for**19:** $W_{g+1}$← $\sum_{k=1}^{k}\frac{\mathrm{nk}}{n}$ $W_{g+1}$**20:** Where: n= $\sum_{k}\mathrm{nk}$**21:** W ← $W_{g+1}$**22:** Node1: publish updated global W to IDSFChain**23:**End for**24:**End procedure**25:**Procedure Edge_Nodes_Update (N, W):**26:**M ← (split data n_k_ into batches of size M)**27:**// train local models at the edge in feed-forward propagation **28:**For each local epoch C = 1, 2, … do**29:**// update local weights in back propagation using stochastic gradient descending (SDG) **30:** W ← W − η ∇ $f_{k}$(W)**31:** Where: ∇ $f_{k}$(W) is the average gradient on edge local data**32:**End for**33:**Node_N_: publish updated local w to IDSFChain**34:**End procedure

### 2.2. Detection Model Description

**Figure 4:**
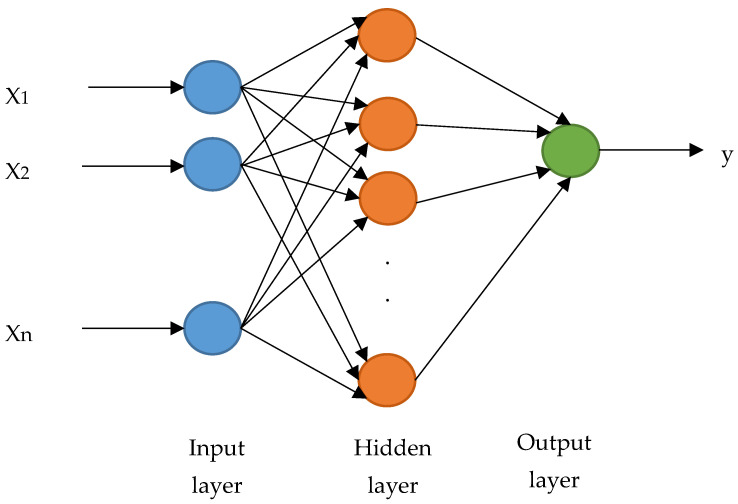
Artificial neural network architecture (binary classification).

ANN is a parallel, distributed system inspired by the biological brain [39]. The most common paradigm of ANN is the multilayer perceptron (MLP) [40]. For the proposed IDS, an ANN has been used. For training, a back propagation algorithm is used at the feed-forward neural network using a BoT-IoT dataset, as shown in Figure 4. Table 1 lists the hyper-parameters used in the proposed detection model.

In our test scenarios, there are some assumptions in the proposed system:

- The same structure and hyper-parameters for all local models, but they are trained with different partitions of the origin dataset.
- The same initial weights for all the clients.
- The weight updates are published to the clients synchronously and regardless of their participation in the last global epoch.
- Common learning rate to all the clients.

## 3. Results

Here, the FIDChain algorithm will be evaluated with ANN and compared with XGBoost using a BoT-IoT dataset, then comparing results with related work tested the same dataset. After evaluation, the algorithm will be tested on different datasets.

### 3.1. Working Environment

The simulation of the FIDChain system was performed on a machine with the following characteristics:

#### 3.1.1. Hardware Characteristics

CPU Intel Xeon 6th generation (1 socket, 8 cores, 16 threads), RAM 32 GB, GPU NVidia Quadro P3000 with cuda v8.0.

#### 3.1.2. Software Characteristics

Keras and PyTorch python ML and FL libraries were used, alongside TensorFlow as a backend engine.

### 3.2. Data Preprocessing

The preprocessing steps of the dataset are performed as follows:

- Removing nominal features and excessive network traffic information by dropping their columns.
- Replacing Null/NaN values with mean or median values.
- Using LabelEncoder function in scikit learn library to encode nonnumeric or symbolic labels into numeric values between 0 and n_classes-1 to be appropriate for learning and testing the proposed model.
- For binary classification, normal and attack traffic attributes were labeled to 0 and 1, respectively.
- Normalizing high-dimensional features using MinMaxScalar function in scikit learn library to a range of (0, 1) to retain feature’s original distribution.
- Dividing dataset into five smaller client datasets to simulate data of five edge devices (acting as gateways for the monitored systems). The dataset was partitioned in such a way that each client with local model can recognize anomalous traffic or intrusions.
- Random splitting of processed dataset into training set (80%) and testing set (20%), knowing that there is no duplication between the testing and the training traffic.

### 3.3. BoT_IoT Dataset

#### 3.3.1. Dataset Description

The BoT-IoT dataset was used to evaluate the performance of the proposed model. It is an IoT traffic-based dataset that was created by designing a genuine testbed environment in the Cyber Range Lab of UNSW. The Canberra Testbed was set up with simulated and actual IoT normal and botnet attacks traffic, which provides more than 73 million records, containing 46 features in each raw, and is provided in csv format of about 16.7 GB in size [41]. The dataset contains normal instances and three types of categorized attacks as follows: information gathering, DoS, and information theft, with their further subcategories to form 10 types of attacks. To ease the training and testing processes of the proposed model, a smaller set (5%) of the original full dataset was extracted and provided by the authors of the dataset. This 5% dataset is comprised of four files with approximately 3 million records and about 1.07 GB total size [42].

#### 3.3.2. Feature Selection

The study [41] provided the selection of the best 10 features of the BoT-IoT dataset (“seq”, “DstIP”, “srate”, “SrcIP”, “max”, “mean”, “stddev”, “min”, “state_number”, and “drate”) using Equation (1) for joint entropy and correlation coefficient lows, which enhances the performance of the proposed IDS and improves accuracy, as shown in Table 2.

(1) $$\mathrm{Entropy}=-\sum_{x}\sum_{y}(p\left(x,y\right)*\log p\left(x,y\right))\;$$

Higher values of entropy refer to lower information gain and depicts randomness of the data. First, the pairwise Shannon joint entropy was calculated using Equation (1), producing *n × n* table, where n is the number of features [43]. Then a score value per feature was introduced through calculating the average joint entropy, then normalizing scores. For measuring the strength of the relationship between the features of the dataset, first the Pearson correlation coefficient is used, producing a matrix. The output of correlation ranges between [−1, 1], and its magnitude indicates the strength of correlation between two feature vectors. Second, the average correlation for each feature of the dataset is calculated and normalized between [0, 1]. The feature with large average joint entropy and low average correlation score was considered ideal.

**Figure 5:**
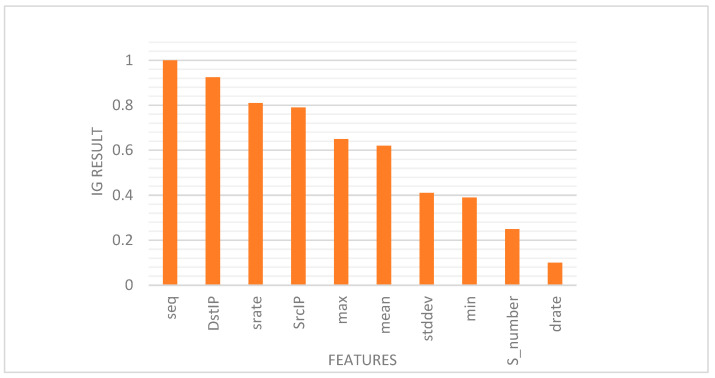
Feature ranking based on information gain.

To reveal the importance of best 10 BoT-IoT dataset features, the adoption of these features with the information gain (IG) was calculated using Equation (2). The most distinguishing features are (‘seq’, ‘DstIP’, ‘srate’, ‘SrcIP’, and ‘max’), while the remaining features that have smaller information gain and lesser contribution to the IDS are (‘mean’, ‘stddev’, ‘min’, ‘state_number’, and ‘drate’), as shown in Figure 5.

(2) $$\mathrm{IG}\left(S,Q\right)=E\left(S\right)-\sum_{i=1}^{K}P_{i}\;E\left(S,Q_{i}\right)$$

### 3.4. Evaluation Methodology of Detection Model

In the classification of attack detection, the FIDChain detection model was evaluated in terms of accuracy, detection rate, precision, recall, specificity, F1-score, and false alarm rate. For binary classification, here are the important metrics to assess the performance of the model.

**Figure 6:**
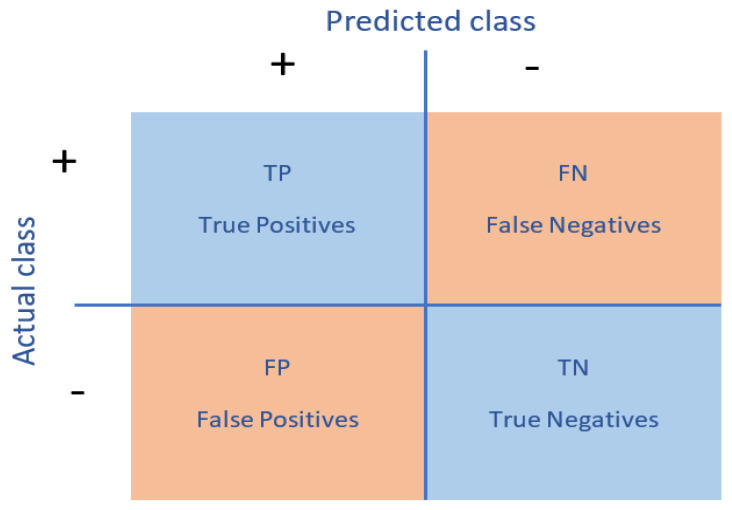
Confusion matrix.

The confusion matrix: this is commonly used to give a more complete picture when evaluating the performance of a model [44], as shown in Figure 6.

The main metrics: this is commonly used to test the performance of classification models by how effective the detection model is in distinguishing between the different classes of network traffic [45]. Table 3 shows the metrics used in this study.

### 3.5. Testing FIDChain Algorithm with BoT-IoT Dataset

**Figure 7:**
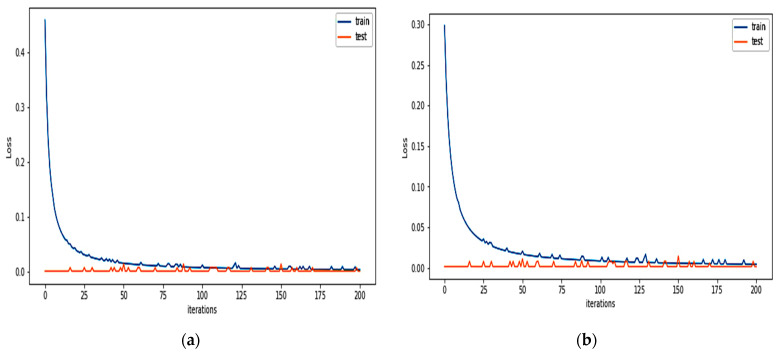
The average of training and testing losses of edge gateways (clients) of the proposed algorithm using: (**a**) BoT-IoT (full features); (**b**) BoT-IoT (best features).

**Figure 8:**
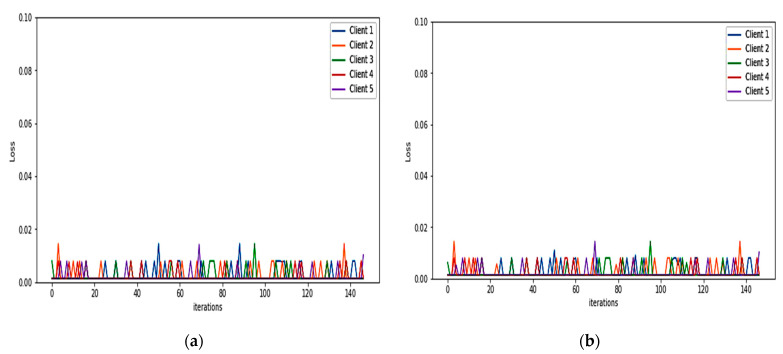
Losses in testing for each client of the proposed algorithm using: (**a**) BoT-IoT (full features); (**b**) BoT-IoT (best features).

For binary classification, the detection model of the FIDChain will be evaluated in terms of accuracy, precision (detection rate), recall (sensitivity), specificity, F1-score, and false alarm rate. In our test scenarios, the BoT-IoT dataset [42] was divided into five smaller client datasets to simulate data of five edge nodes (clients). The detection model has been tested on two ML models, ANN and XGBoost, using a dataset with “Full Features” and “Best 10 Features”. Table 4 presents the obtained results. Figure 7a,b show the average of training and testing losses of edge gateways (clients) of the proposed algorithm with ANN with the BoT-IoT dataset (full features, best features respectively), there is a good cutoff point for the loss which mostly occurs after about 150 communication rounds (iteration). Figure 8a,b show losses of testing for each client individually. 

The results show that ANN has better performance than XGBoost because of higher accuracy in both dataset versions (99.99%), whereas XGBoost has less accuracy of 98.4% and 98.96% for the full features version and best features version of the dataset, respectively. These results are obtained by taking the average results obtained from all edge local models. ANN has a higher detection rate (100% for BoT-IoT dataset with full features and best features) than XGBoost (99.36% and 99.38% for BoT-IoT dataset full features and best features, respectively), which indicates the accurate positive predictions of attacks. This implies that our proposed solution can minimize the false positives. ANN detection models have higher coverage of actual positive sample with a 99.99% recall result for the dataset than XGBoost with 99.59% and 99.57% for BoT-IoT dataset with full features and best features, respectively. The F1-score of ANN is better than that of XGBoost (99.99% and 99.47% for the BoT-IoT dataset with full features and best features, respectively). The F1 scores show that ANN performs better in considering both false positives and false negatives. Furthermore, ANN has better specificity than XGBoost, which indicates better coverage of actual negative instances. Additionally, ANN has lower false alarm rate than XGBoost, which indicates lower false predictions of the attacks.

Finally, by examining the results it is obvious that the proposed FIDChain with ANN detection model is much better than using other recent methods, such as XGBoost. This is because ANNs have many advances that make it better and more suitable for intrusion detection. ANN has the following qualities: the ability to learn complex and nonlinear relationships as in IoT applications [46]; it can generalize and predict unseen data after learning from the relationships of the initial data; it does not place any restrictions on the dataset and its distribution; and it has better performance with the heterogeneity of data in IoT devices, such as in ICUs in healthcare systems. The FIDChain mechanism is suitable for healthcare systems because it has the ability to quickly and accurately predict the intrusions and attacks, is simpler, and is suitable for edge-distributed models with the limited processing capacity of healthcare IoT devices.

### 3.6. Evaluating Blockchain with the Federated System

**Figure 9:**
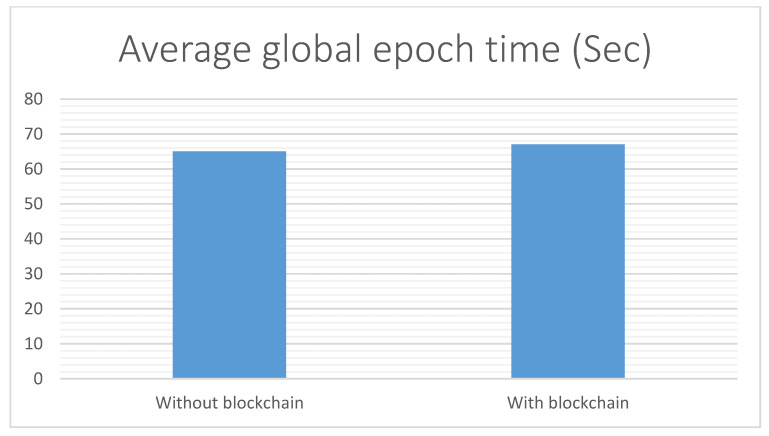
Average global epoch time with and without blockchain.

The public blockchain that depends on the proof of work consensus protocol will not be feasible for the federated learning process as it involves a large amount of weight updates. The generation of new blocks on the ledger would be too slow such as in Hyperledger or Ethereum. Therefore, the proposed system relies on a private and permissioned blockchain that supports a consensus algorithm based on block signatures and a customizable round-robin consensus scheme without proof of work. The blockchain algorithm increases the complexity with limited impact on the federated learning performance. At the end of each local update, the edge node writes the weights event on the blockchain and the CPCC computes the weighted average of the local weights. However, as most blockchains create new blocks at fixed time intervals, we propose to line up the averaging process with the period of the block creation to minimize any latencies. The duration of training on a single edge node was approximately 54 min with up to one minute more or less on average, i.e., 54 ± 1. We have noticed there is some execution time complexity because of the blockchain algorithm increases the complexity with limited impact on the federated learning time. The impact can be measured experimentally before and after the activation of blockchain. The approximate time loads per epoch are calculated with and without using blockchain. From Figure 9, the overhead of about 3% is estimated (2 s at average per epoch), while providing full transparency and immutability over the distributed training process.

### 3.7. Comparison of FIDChain with the State of the Art

Here, we compare the proposed FIDChain algorithm with the related work that uses some popular ML and DL algorithms tested on the BoT-IoT dataset, as shown in Table 5. The average accuracy, recall, and F1-score of the FIDChain is superior to those of the other methods in most classes with the highest precision, which means that the proposed FIDChain can minimize the false positives in comparison with the stated approaches. The study [47] used the advances of blockchain as storage with 99.99% accuracy, but the learning IDS is centralized, which compromises medical data. The study [48] proposed an algorithm that provides an IDS with auto-encoder model and uses the advances of blockchain to store local weights, tacking average and providing updated weights. For evaluation, the CICIDS2017 dataset was used and the obtained accuracy was 97%, which is lower than FIDChain. However, it deletes the centralized server from the blockchain network, which leads to no update of the global model weights. The study [38] proposed an algorithm that presents an IDS in FL mode with ANN, but with lower accuracy (92.5%) than FIDChain, and it did not make use of blockchain advances of protecting from poisoning attacks.

### 3.8. Testing FIDChain with ANN Using Different Data Sets

**Figure 10:**
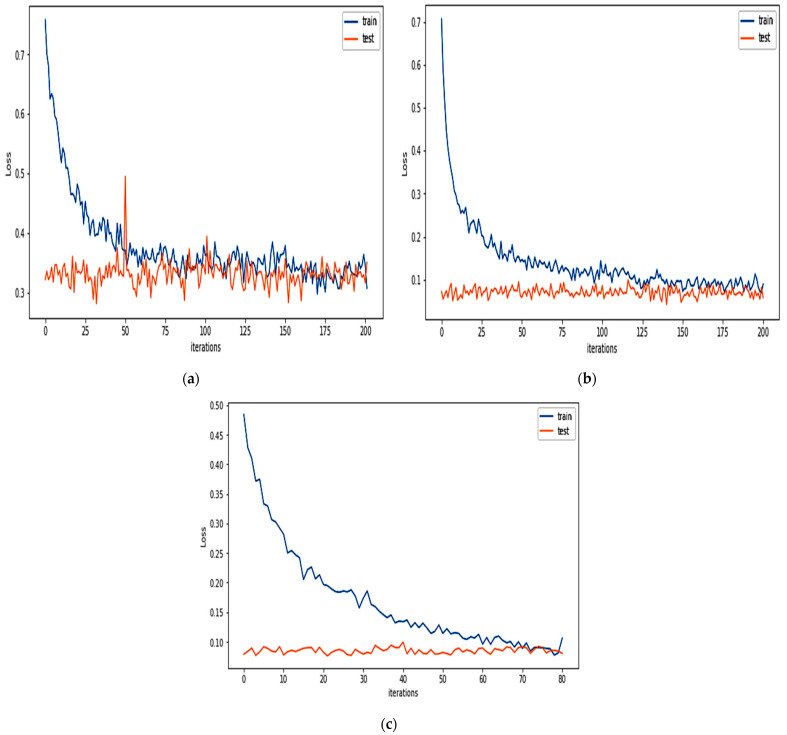
The average of training and testing losses of edge gateways (clients) of the proposed algorithm using: (**a**) CSE-CIC-IDS2018; (**b**) Bot Net IoT; and (**c**) KDD Cup 99.

**Figure 11:**
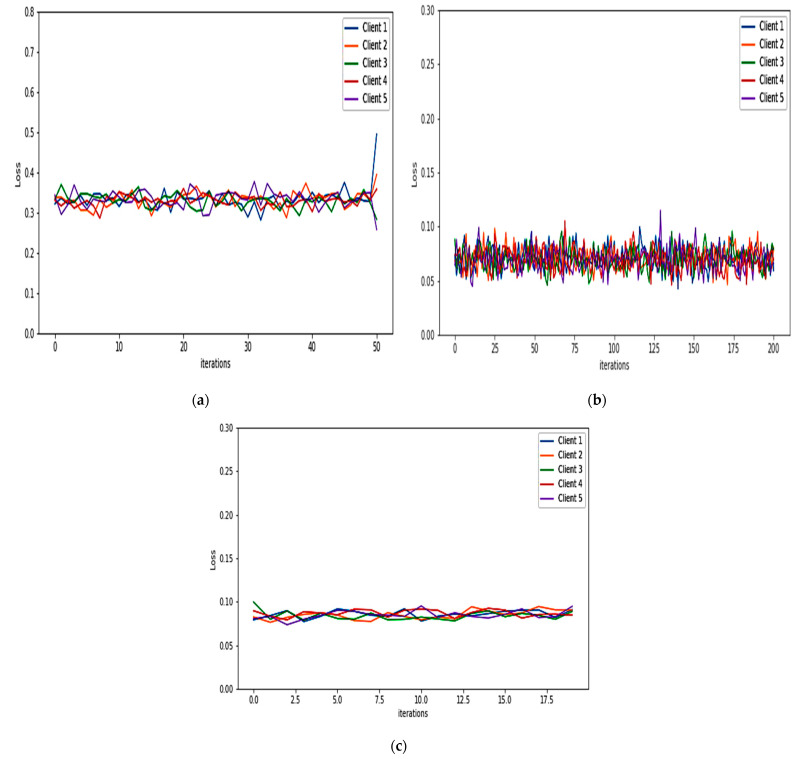
Losses in testing for each client of the proposed algorithm using: (**a**) CSE-CIC-IDS2018; (**b**) Bot Net IoT; and (**c**) KDD Cup 99.

Here, the performance indicators of the proposed FIDChain will be evaluated in terms of precision (detection rate), recall (sensitivity), F-score, specificity, accuracy, and false alarm rate using different datasets and compared to the BoT-IoT (full and best features). Table 6 describes these datasets. The datasets were prepared with the same test scenario as BoT-IoT by dividing them into five smaller client datasets to simulate data of five edge nodes (clients). Table 7 shows the obtained results, which reveal that the BoT-IoT dataset gives the most stable and accurate results, as it is the IoT traffic-based dataset that has more variety of botnet and it is the most suitable for testing IoT applications, such as in healthcare systems. Figure 10a–c show the average of training and testing losses of edge gateways (clients) of the proposed algorithm using CSE-CIC-IDS2018, Bot Net IoT, and KDD Cup 99 datasets, respectively. Figure 11a–c show losses of testing for each client individually.

## 4. Conclusions

In this paper, an edge-cloud intrusion detection mechanism was introduced with the integration of blockchain distributed ledger, FIDChain, which is simple and suitable for deployment on edge devices in healthcare systems. These healthcare systems use IoT devices with limited storage capacity and lower computations. FIDChain has the ability to detect multiple botnets faster as the detection model is trained in the edge server so as to protect the cloud or other resources if a particular attack happens, as it blocks the data from its gateway, with smaller detection time as the attack resources become nearer. The proposed FIDChain undergoes lesser computing and processing capacity because the FL model deals with smaller sets of data. Furthermore, the proposed FIDChain model provides a solution to poisoning attacks, which is one of the most important challenges facing FL, as it uses the blockchain network to store the weights of each local model in a distributed ledger in chained blocks. These chained blocks connect gateway nodes with server node in the next cloud layer, which is further used for aggregation and averaging purposes. Finally, the chain is protected using a cryptographic hash function that connects the blocks together in the chain. Thus, it cannot be tampered with or changed, as it operates on a smart contract. Although that blockchain increases the complexity of the system, it had a limited impact with a negligible time overhead of about 3% per epoch in terms of providing full transparency and immutability over the distributed system. After evaluation in terms of different performance indicators, such as precision (detection rate), recall (sensitivity), F-score, specificity, accuracy, and false alarm rate, using real IoT traffic-based dataset ‘‘BoT-IoT”, the results obtained show that FIDChain not only enhances the accuracy (99.99%) and false alarm rate but also outclasses the latest ML and DL models. In addition, it gives balanced results of the intrusion detection. For further evaluation, the FIDChain with ANN has been tested with different datasets: CSE-CIC-IDS2018, Bot Net IoT, and KDD Cup 99. The obtained results show that the BoT-IoT dataset gives the most stable and accurate results, as it is the IoT traffic-based dataset that has more variety of botnet types, and it is the most suitable for testing IoT applications, such as in healthcare systems. In future work, the performance of the proposed FIDChain should be improved using multiple classifications to detect the type of attack and trace its source for further enhancement of protecting healthcare systems; the blockchain-based federated learning solution would be applied to more use cases with different neural network architectures, the algorithm would be improved to deal with large number of heterogeneous hardware, and the aggregation algorithm would be enhanced to deal with the delay caused by the slowest contributor.

## Figures and Tables

**Table 1:** The hyper-parameters used in the proposed detection model.

Hyper-Parameters	Value
Learning rate	0.001:0.1 (+0.01)
Number of epochs	2:10 (+1)
Batch size	100:1000 (+100)
Classification type	Binary
Activation function	Sigmoid
Optimization algorithm	Stochastic gradient descent (SGD)

**Table 2:** The description of BoT-IoT best features.

State_Number	Numerical Representation of Feature State
Seq	Argus sequence number
N_IN_Conn_P_SrcIP	Number of inbound connections per source IP
N_IN_Conn_P_DstIP	Number of inbound connections per destination IP
Srate	Source-to-destination packets per second
Drate	Destination-to-source packets per second
Min	Minimum duration of aggregated records
Max	Maximum duration of aggregated records
Mean	Average duration of aggregated records
Stddev	Standard deviation of aggregated records

**Table 3:** Effectiveness main metrics.

Metric	Equation	Definition
Accuracy	$\frac{\mathrm{TP}+\mathrm{TN}}{\mathrm{TP}+\mathrm{TN}+\mathrm{FP}+\mathrm{FN}}$	Ratio of correctly predicted instances to total number of predicted instances.
Precision (Detection rate)	$\frac{\mathrm{TP}}{\mathrm{TP}+\mathrm{FP}}$	Ratio of the correctly predicted positive instances to total positive predictions.
Recall (Sensitivity)	$\frac{\mathrm{TP}}{\mathrm{TP}+\mathrm{FN}}$	Ratio of the correctly predicted positive instances to the overall available positive data category.
Specificity	$\frac{\mathrm{TN}}{\mathrm{TN}+\mathrm{FP}}$	Ratio of the correctly predicted negative instances to the overall available negative data category.
F1-score	$\frac{2\mathrm{TP}}{2\mathrm{TP}+\mathrm{FP}+\mathrm{FN}}$	Hybrid metric indicates the overall performance of the model respecting to both precision and recall, useful for unbalanced classes
False alarm rate	$\frac{\mathrm{FP}}{\mathrm{FN}+\mathrm{FP}}$	Ratio of false positive alarms per the total number of false prediction warnings or alarms.

**Table 4:** The performance analysis of FIDChain using ANN compared to XGBoost with BoT-IoT dataset.

ML Algorithm	ANN		XGBOOST	
Dataset Version	Full Features	Best 10 Features	Full Features	Best 10 Features
Accuracy	99.99%	99.99%	98.40%	98.96%
Precision (Detection Rate)	100%	100%	99.36%	99.38%
Recall (Sensitivity)	99.99%	99.99%	99.59%	99.57%
F-score	99.99%	99.99%	99.47%	99.47%
Specificity	88.89%	100%	56.98%	57.12%
False Alarm Rate	11.11%	0%	43.02%	42.88%

**Table 5:** Comparison with related work tested on BoT-IoT dataset.

Ref.	Model	Classification Type	Accuracy	Precision (Detection Rate)	Recall	F1-Score	Mode	Integration with Blockchain
[49]	CNN-TSODE	Binary	99.99%	99.99%	99.99%	99.99%	Centralized	No
		Multi	99.04%	99.04%	99.04%	99.04%		
[50]	DNN	Multi	98.37%	-	-	-	Centralized	No
	RNN							
	CNN							
[51]	RNN	Multi	98.20%	-	-	-	Centralized	No
[37]	DeepDCA (DCA-SNN)	Binary	98.73%	99.17%	98.36%	98.77%	Centralized	No
[52]	Naive Bayes	Binary	51.5%	-	-	-	Centralized	No
	KNN		92.1%	-	-	-		
	ANN		82.8%	-	-	-		
[47]	RF	Multi	99.99%	99.99%	99.99%	99.99%	Centralized	Yes
	XGBoost		99.99%	87.77%	94.36%	87.90%		
[53]	NB	Binary	52.18%	79.67%	99.70%	69.50%	Centralized	No
	KNN		99.48%	99.65%	99.68%	99.58%		
	RF		99.51%	99.70%	99.79%	99.65%		
	Log R		99.50%	95.28%	90.39%	94.70%		
	DT		99.47%	99.69%	99.79%	99.63%		
[54]	decision tree	Multi	99.99%	97.10%	94.27%	98.95%	Centralized	No
	Naive Bayes		97.49%	56.28%	57.95%	98.44%		
	Random Forest		99.98%	95.05%	91.37%	99.99%		
	SVM		97.80%	57.89%	43.24%	98.48%		
[38]	ANN	Multi	99.9%	-	-	-	Centralized	No
			92.5%	-	-	-	Federated	
Our work	ANN	Binary	99.99%	100%	99.99%	99.99%	Federated	Yes

**Table 6:** Description of used datasets.

Dataset	Description
CSE-CIC-IDS2018 [55]	Network traffic-based dataset proposed by the Communications Security Establishment (CSE) & the Canadian Institute for Cybersecurity (CIC) including 7 botnet types with 80 network flow features.
Bot Net IoT [56]	Internet-connected devices-based dataset proposed by Beigi et al. which is divided into training (with 7 botnet types) and test datasets (with 16 botnet types) with four groups of features (byte-based, packet-based, time, and behavior-based).
KDD Cup 99 [57]	Network traffic-based dataset consists of approximately 4,900,000 vectors. The botnet types are divided into four categories (user-to-root attack (U2R), remote-to-local attack (R2L), probing attack, and denial-of-service attack (DoS)) containing 41 features, which are categorized into three classes (basic features, traffic features, and content features).

**Table 7:** Results of testing FIDChain with different datasets.

Dataset	Precision (Detection Rate)	Recall (Sensitivity)	F-Score	Specificity	Accuracy	False Alarm Rate
CSE-CIC-IDS2018	0.4461	0.8581	0.5870	0.8589	0.8588	0.1411
Bot Net IoT	1.0000	0.9742	0.9869	0.9996	0.9756	0.0004
Bot-IoT (10 Features)	1.0000	0.9999	0.9999	1.0000	0.9999	0.0000
Bot-IoT (All Features)	1.0000	0.9999	0.9999	0.8889	0.9999	0.1111
KDD Cup 99	0.9709	0.9491	0.9599	0.9928	0.9840	0.0072

## Data Availability

All datasets used in this research are available online for the research community, The BoT-IoT Dataset: https://www.unsw.adfa.edu.au/unsw-canberracyber/cybersecurity/ADFA-NB15-Datasets/bot_iot.php (accessed on 4 May 2021), CSE-CIC-IDS2018 Dataset: https://www.unb.ca/cic/datasets/ids-2018.html (accessed on 8 December 2021), Botnet Dataset: https://www.unb.ca/cic/datasets/botnet.html (accessed on 23 October 2021), Kdd Cup 1999: http://kdd.ics.uci.edu/databases/kddcup99/kddcup99.html (accessed on 30 May 2021).
